# Prevalence, risk factors, and perceptions of vaccination against reproductive tract infections among urban females in Delhi: a cross-sectional study

**DOI:** 10.3389/frph.2026.1812966

**Published:** 2026-05-26

**Authors:** Priya Bhardwaj, Sunita K. Yadav, Joyeta Ghosh, Ravi Kant, Anita Garg Mangla, Sumathi Muralidhar, Daman Saluja, Aleksandra E. Sikora, Jyoti Taneja

**Affiliations:** 1 Department of Pharmacology, All India Institute of Medical Sciences, New Delhi, India; 2 Laboratory of Reproductive Epidemiology and Infection Biology, Department of Zoology, Daulat Ram College, University of Delhi, Delhi, India; 3 Department of Dietetics and Applied Nutrition, Amity Institute of Applied Sciences (AIAS), Amity University - Kolkata Campus, Kolkata, West Bengal, India; 4 Computational Drug & Vaccine Discovery Laboratory, Faculty of Applied Sciences and Biotechnology, Shoolini University, Solan, Himachal Pradesh, India; 5 Department of Biochemistry, Daulat Ram College, University of Delhi, Delhi, India; 6 Apex Regional STD Centre, Safdarjung Hospital, New Delhi, India; 7 Dr. B.R. Ambedkar Center for Biomedical Research, University of Delhi, Delhi, India; 8 Department of Pharmaceutical Sciences, College of Pharmacy, Oregon State University, Corvallis, OR, United States; 9 Vaccine and Gene Therapy Institute, Oregon Health & Science University, Beaverton, OR, United States

**Keywords:** menstrual hygiene, reproductive tract infections (RTIs), RTI prevalence, sexually transmitted infections (STIs), vaccine hesitancy

## Abstract

**Background:**

Reproductive tract infections (RTIs) including sexually transmitted infections (STIs), endogenous and iatrogenic infections, are a major health concern globally, particularly among young females in developing nations. If untreated, they can lead to infertility, increased HIV susceptibility, and cervical cancer. This study examines prevalence of self-reported RTI symptoms, reproductive health status, health literacy, and associated risk factors and perception towards emerging STI vaccines among females in an urban setting.

**Methods:**

A cross-sectional study was conducted among 1,935 urban females, predominantly aged 18–25 years, from non-medical colleges located in Delhi using a structured questionnaire. Data from 15 participants were excluded due to incomplete information. The remaining data (*N* = 1,920) was analyzed using SPSS 20 following Chi-square tests, Fisher's exact tests, and Bonferroni corrections. Multinomial logistic regression (reference category: RTI-absent) identified risk factors for low- and high-risk RTIs. Additionally, a binary logistic regression was conducted to identify risk factors of STI/RTI vaccine acceptance.

**Results:**

Among total respondents, 1,241(64.6%) self-reported RTI symptoms, with 1,029 (83%) experiencing vaginal discharge and 974 (78.5%) vulval itching. Significant risk factors among high risk-RTIs, included, menstrual hygiene practices (OR = 3.183), awareness of contraceptive methods (OR = 1.919), family history of medical conditions (OR = 1.494) and prior RTI medication use (OR = 8.839). While, significant risk factors among low risk-RTIs, included, awareness of contraceptive methods (OR = 1.475), and prior RTI medication use (OR = 10.642). Approximately 8.2% and 25.9%, had been vaccinated against HPV and hepatitis B, respectively, while 54.3% and 79.5% of respondents were aware of these vaccines, respectively. Additionally, 1032 (53.8%) were hesitant about emerging RTI vaccines, citing safety concerns (36%, *n* = 372) and cost (18.6%, *n* = 192). However, 42.9% (*n* = 826) of participants reported that recommendations by healthcare providers positively influenced their willingness to receive vaccination.

**Conclusion:**

The high prevalence of self-reported RTI symptoms highlights the urgent need for screening, healthcare services, literacy and awareness program to enhance RTI prevention and management.

## 1 Background

Reproductive Tract Infections (RTIs) refer to infections affecting the reproductive system in both men and women. These infections are broadly categorized into three types: sexually transmitted infections (STIs), endogenous infections, and iatrogenic infections. RTIs are a major public health concern, particularly for females, as they can significantly impact reproductive health, pregnancy outcomes, and overall well-being (1–3). Often described as a “silent” pandemic, RTIs have far-reaching consequences not only for women's sexual and reproductive health but also for their families and communities worldwide. Females of reproductive age are particularly vulnerable to RTIs (1), especially during pregnancy (2), menstruation (3, 4), and childbirth (2).

Common symptoms of RTI include lower abdominal pain, backache, abnormal vaginal discharge, vulvar itching, and genital ulcers (2, 3, 5). Timely treatment is crucial, as untreated RTI can lead to severe complications such as ectopic pregnancy, infertility, cancer, neonatal mortality, and an increased risk of HIV transmission (3, 6–9). Despite these risks, RTI remains widely underreported in developing countries due to stigma, low awareness, and inadequate healthcare services (3, 10). In India alone, approximately 6% of the adult population is estimated to have one or more RTI/STIs, contributing to an annual burden of 30–35 million cases (11). A previous study in Northern India reported a 9.7% prevalence of RTI diagnosed through laboratory tests in women attending a tertiary care center (12). In another community-based study conducted in Delhi, 53.7% of women self-reported RTI/STI, with 36.7% confirmed by laboratory diagnosis (13).

The World Health Organization (WHO) estimates that approximately 374 million new cases of curable STIs occur annually among individuals aged 15–49 years, including gonorrhea, chlamydia, syphilis, and trichomoniasis (3, 10). However, these estimates exclude viral STIs such as human immunodeficiency virus (HIV), hepatitis B, genital warts, and genital herpes (12). Additionally, bacterial vaginosis (BV) has emerged as a significant RTI, characterized by an imbalance in the vaginal microbiome that increases susceptibility to other infections (14). Given the overwhelming prevalence of RTIs, strengthening epidemiological data collection is essential for effective management and control (3). To address this, WHO recommends four key surveillance methods at the country level: case reporting, etiological analysis, monitoring of antimicrobial resistance, and prevalence assessment (3, 15). In resource-limited settings where diagnostic facilities are inaccessible, syndromic case management based on identifying signs and symptoms rather than laboratory diagnosis remains the primary approach for RTIs control (3, 16).

Despite advancements in healthcare, the persistently high prevalence of RTI underscores the need for evidence-based interventions targeting key factors driving disease transmission. Vaccines offer a promising preventive strategy, as demonstrated by the success of human papillomavirus (HPV) and hepatitis B vaccines, which have significantly reduced genital tract infections and related complications (3, 17). However, despite their potential in reducing STI prevalence, public acceptance of STI vaccines remains a major challenge due to misinformation and safety concerns.

Furthermore, despite availability of substantial research on the epidemiology of RTIs (18, 19), important gaps remain in understanding the intersection between disease burden, health literacy, and perceptions toward emerging STI vaccines, particularly in urban populations. Vaccine hesitancy in the context of STIs is influenced by socio-cultural stigma, misinformation, and safety concerns, and remains inadequately explored. Furthermore, healthcare provider recommendation has been consistently identified as a key determinant of vaccine acceptance. Addressing these gaps is essential for designing targeted interventions to improve reproductive health outcomes and vaccine uptake. Therefore, the primary objective of the study was to assess the prevalence of self-reported reproductive tract infections (RTIs), reproductive health status, health literacy, and associated risk factors among urban females. In addition, the study evaluated perceptions regarding STI vaccines and the factors contributing to vaccine hesitancy among urban females.

## 2 Materials and methods

### 2.1 Study design

A cross-sectional study was conducted among urban females (18–50 years). The questionnaire was developed by the corresponding author and co-authors, with its basic framework adapted from the World Health Organization (WHO) guidelines (20) and relevant literature (13, 21–23). Inputs were also provided by co-authors, including a microbiologist, a senior professor with expertise in sexually transmitted diseases (STDs), and a gynecologist. Based on the societal context, the questionnaire was expanded and written in simplified language to facilitate symptom-based diagnosis and improve understanding among the target population. Initial validation was performed through a face-validity pilot study, after which the expert-reviewed questionnaire was used for the full-scale study (Supplementary File S1). The questionnaire also covered demographics, reproductive health, menstrual hygiene practices, health literacy, comorbidities and perception towards emerging RTI vaccines.

### 2.2 Methodology

The questionnaire was pre-designed, pre-tested, structured, and closed-ended, and was administered online through Google Forms in the English language. It was conducted across four non-medical colleges of University of Delhi, two from the South Campus and two from the North Campus, offering streams in Science, Commerce, and Humanities. Participants included students (18–25 years), and faculty members, representing diverse backgrounds and regions, including Delhi/NCR (*N*ational Capital Region) and other parts of India.

An awareness program on sexual and reproductive health was organized at the host non-medical college to introduce the study objectives. During the session, a gynecologist and clinicians with expertise in STDs guided participants in identifying symptoms and understanding their association with reproductive tract infections (RTIs). The program was attended by students and faculty members of host and participating colleges. Faculty members interested in study objectives were provided with a detailed explanation of each questionnaire item. Subsequently, these trained faculty members facilitated questionnaire administration within their respective colleges to ensure accurate responses, while also addressing participants’ queries.

#### 2.2.1 Inclusion criterion

- The online questionnaire was administered to females aged 18 and above (maximum 50 years).
- The first question in the Google questionnaire was participants’ consent to take part in the study, only those who selected “Yes” could access the rest of the questionnaire form.
- All the participants were assured of anonymity and confidentiality of their identities.
- Only those who provided complete information in the Google Form were included in the study.

#### 2.2.2 Exclusion criterion

- Participants younger than 18 years were excluded from the study.
- Those who did not provide consent and complete information in the Google Form were also excluded.

#### 2.2.3 The sample size formula used for calculating the sample size is given below

A prevalence of STI/RTIs as 36.8% (24), relative precision of 10%, and an alpha error of 0.05 and accounting for a non-response rate of 10%, the final sample size came out to be 733. To account for the higher number of variables (RTIs symptoms=5), double the sample size (*N* = 1,466) for survey data collection was taken. The sample size formula used for calculating the sample size is given below:

$$n\;=Z_{*}^{2}\;p_{*}(1-p)/d^{2}$$

- Assumed prevalence *p* = 0.368, *p* = 0.368 (36.8%), Relative precision r = 10%, absolute precision d = r × *p* = 0.10 × 0.368 = 0.0368
- Confidence level 95% → Z_0.975_ = 1.96 = 1.96^2^ × 0.368 × (1–0.368)/0.368^2^ = 659.75 = 660
- Non-response (e.g., 10%): 660/0.90 = 733

### 2.3 Participant classification

A total of 1,935 responses were collected from urban females. Of these, 15 responses were excluded due to incomplete information, leaving 1,920 responses for analysis. Among the 1,920 analyzed responses, 679 (35.4%) were RTI-absent and 1,241 (64.6%) were RTI present groups based on self-reported symptoms suggestive of reproductive tract infections (RTIs). This group was further stratified into: 1) Low-risk RTI (*N* = 446, 23.2%)—Participants reporting two or fewer symptoms; 2) High-risk RTI (*N* = 795, 41.4%)—Participants reporting more than two symptoms.

### 2.4 Data analysis

This study adhered to the Strengthening the Reporting of Observational Studies in Epidemiology (STROBE) guidelines to ensure transparent and standardized reporting. The data was pre-processed and analyzed using SPSS 20. The statistical methods applied included descriptive analyses for sociodemographic and categorical variables, as well as Chi-square tests, Fisher's exact tests. A Bonferroni corrections test was used for pairwise comparisons among low-risk RTI, high-risk RTI, and RTI-absent groups, with a significance threshold of 0.0167. Multinomial logistic regression was conducted to identify risk factors for RTI, with the RTI-absent group (*N* = 679) as the reference category. All candidate variables were entered simultaneously using the Enter method, selected a prior based on biological plausibility and the WHO syndromic management framework. The final model was run on the complete analytic sample (*N* = 1,920), with missing data negligible (<0.5% per variable). Multicollinearity was assessed using Variance Inflation Factors (VIF; all <2.3). Model fit was evaluated using the likelihood ratio (LR) chi-square test and Nagelkerke pseudo-R². Additionally, a binary logistic regression was performed to identify risk factors of STI/RTI vaccine acceptance. Sensitivity analyses were conducted by excluding participants who had used RTI/UTI medication in the past three months, and additionally excluding those with a prior RTI diagnosis, to assess the robustness of the menstrual hygiene and contraceptive awareness associations. A 5% significance level was used for all comparisons. The study also assessed participants’ attitudes toward vaccine efficacy, defined as the ability of a vaccine to reduce disease cases compared to no vaccination.

### 2.5 Visualization and illustrations

Graphs were generated using GraphPad Prism 10, and illustrations were created using BioRender to visually represent the study findings.

## 3 Results

### 3.1 Demographic profile of participants

**Figure 1:**
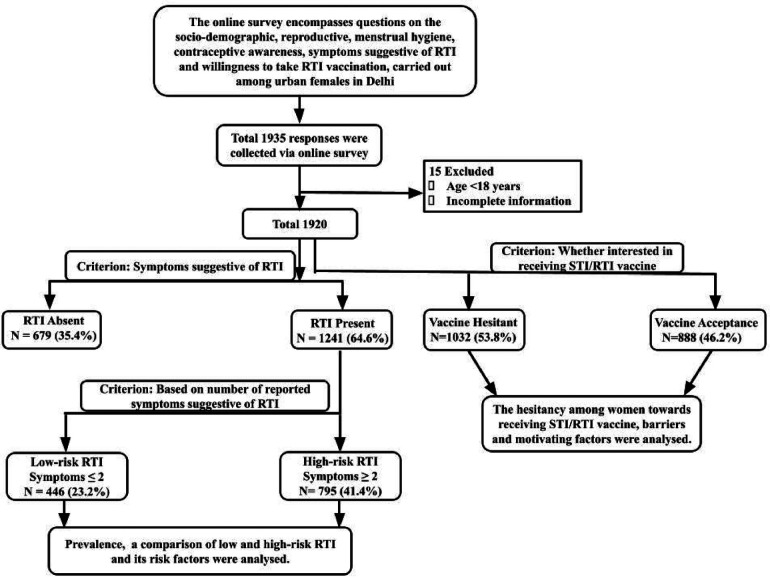
STROBE flow diagram of participant selection and classification. This flow diagram illustrates the selection process and classification of study participants. A total of 1,935 respondents were initially screened. After excluding 15 individuals due to incomplete information or being under 18 years old, 1,920 respondents were included in the final analysis. Participants were categorized based on the presence of reproductive tract infections (RTIs) symptoms and their interest in RTIs/ STIs vaccination.

A flow diagram detailing participant inclusion, exclusion, and classification is presented in Figure 1, following STROBE guidelines. The demographic profile of respondents is presented in Table 1. The majority of participants (94.6%) were between 18 and 25 years old, while the remaining 5.4% were aged 26–50.

**Table 1:** Sociodemographic and health awareness characteristics of study participants (*N* = 1,920).

Characteristics	*N* (%)
Age (Years)	
18–25	1,788 (94.6)
26–50	102 (5.4)
BMI Status	
Underweight (<18.5 Kg/m^2^)	387 (21.9)
Normal weight (18.5–24 Kg/m^2^)	1,104 (62.4)
Overweight (25–29.9 Kg/m^2^)	217 (12.3)
Obese (>30 Kg/m^2^)	62 (3.5)
Marital Status	
Unmarried youth	1,844 (96.0)
Married	76 (4.0)
Education status	
XII Grade	1,342 (69.9)
Graduate	469 (24.4)
Postgraduate and above	109 (5.7)
Occupation	
Students	1,776 (92.5)
Academician/Government/Private/Healthcare workers	110 (5.7)
Home makers	34 (1.8)
Ever suffered from symptoms of RTI	
Yes	1,241 (64.6)
No	679 (35.4)
Understanding of STI	
Poor	332 (17.3)
Moderate	1,167 (60.8)
Good	420 (21.9)
Interest in receiving STI/RTI vaccine	
Yes	888 (46.2)
No	1,032 (53.8)
Heard of HPV vaccines	
Yes	1,043 (54.3)
No	877 (45.7)
Received HPV vaccine	
Yes	158 (8.2)
No	1,761 (91.8)
Heard of Hepatitis B Vaccines	
Yes	1,498 (79.5)
No	386 (20.5)
Received Hepatitis B Vaccine	
Yes	497 (25.9)
No	1,422 (74.1)

BMI, body mass index; RTI, reproductive tract infection; STI sexually transmitted infection.

Among all respondents, 62.4% had a normal BMI (body mass index), while 21.9% were underweight, 12.3% were overweight, and 3.5% were obese.

Regarding marital status, 96% of respondents were unmarried, while the remaining 4% were married. In terms of education, 69.9% had completed up to the twelfth grade, followed by graduates (24.4%) and postgraduates or higher (5.7%). Most of the participants (92.5%) were students, while 5.7% were academician/government/private/healthcare workers and 1.8% were home makers.

Among the total respondents, 64.6% reported experiencing symptoms suggestive of RTI. Therefore, the prevalence of RTI among females in Delhi/NCR was found to be 64.6%. When asked about their understanding of STIs, 60.8% had a “moderate understanding,” 21.9% had a “good understanding,” and 17.3% had a “poor understanding.”

Regarding their willingness to receive vaccines against STIs, 53.8% of participants were hesitant, while 46.2% were willing to get vaccinated. Additionally, 45.7% of participants were unaware of the HPV (human papillomavirus) vaccine, and 20.5% were unaware of the hepatitis B vaccine. However, 8.2% had already received the HPV vaccine, and 25.9% had been vaccinated against hepatitis B (Table 1). Participants were also asked about the age at which they received these vaccines. Among those who had received the HPV vaccine (*N* = 158, 8.2%), only 89 (56.3%) reported their age at the time of vaccination. Most of them (*N* = 70) had received the vaccine between the ages of 11 and 18. Similarly, among the 497 participants who had received the hepatitis B vaccine, 276 (55.5%) reported their age at vaccination. The minimum reported age for receiving the hepatitis B vaccine was below 1 year (*N* = 200), while the maximum was 30 years (*N* = 76).

### 3.2 Prevalence of reproductive tract infections (RTIs)

The prevalence of RTI was assessed using the WHO syndromic method guidelines. During the study, participants were asked whether they had experienced any symptoms listed in the questionnaire. Among all participants, 64.6% (*N* = 1,241) reported at least one symptom suggestive of RTI. Of these, 23.2% (*N* = 446) reported two or fewer symptoms and were categorized as the low-risk RTI group. The remaining 41.4% (*N* = 795) reported more than two symptoms and were classified as the high-risk RTI group (Figure 1).

#### 3.2.1 Frequency of symptoms suggestive of RTI

**Figure 2:**
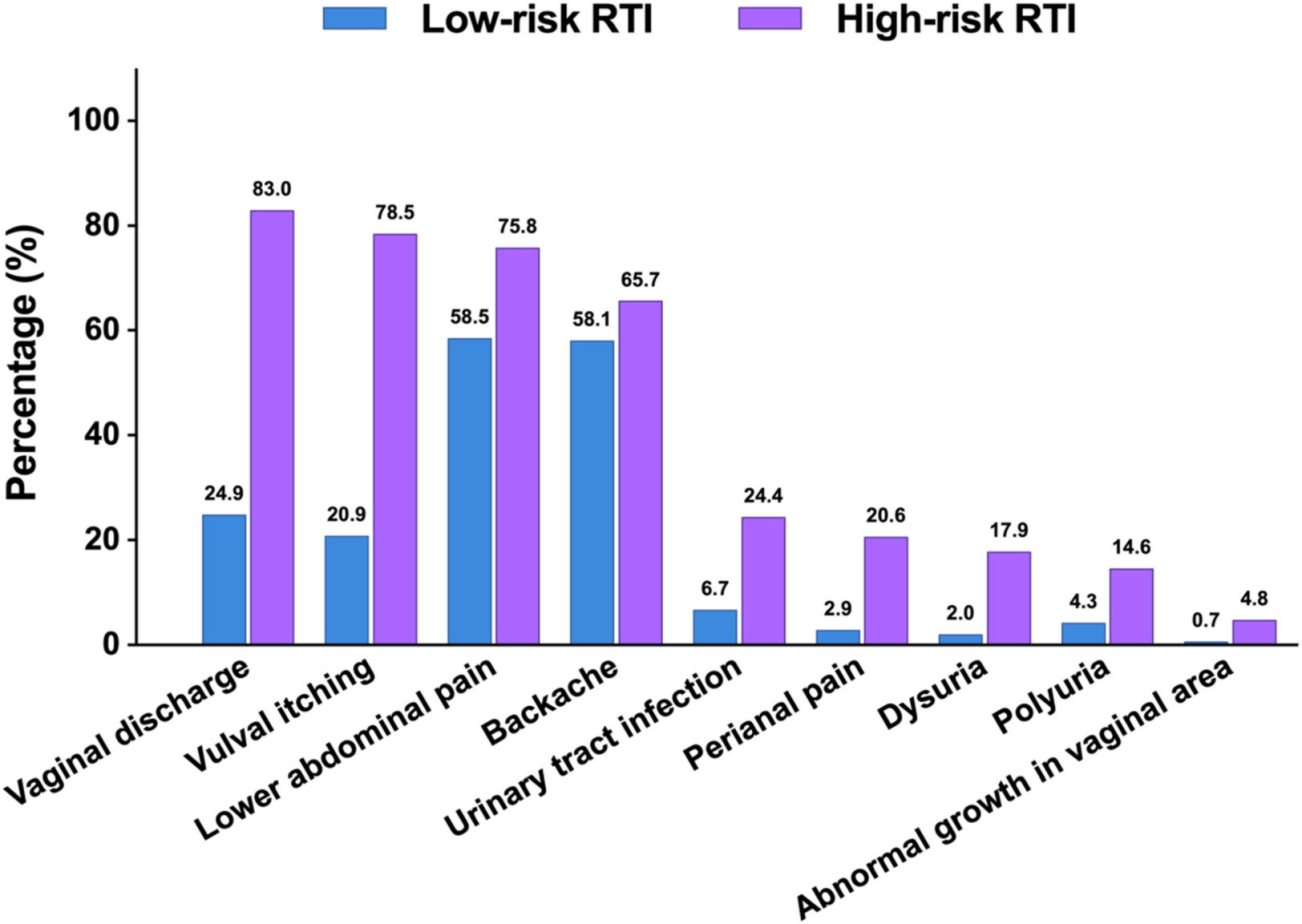
Distribution of symptoms among Low- and high-risk RTI groups. This bar chart presents the prevalence of symptoms associated with reproductive tract infections (RTI) among participants classified as low-risk and high-risk. The *x*-axis represents different RTI symptoms, while the *y*-axis shows the percentage of participants experiencing each symptom. The percentage for each symptom is displayed above its corresponding bar in the group.

The symptoms reported by participants in both low- and high-risk RTI groups are shown in Figure 2. Among high-risk RTI participants, the most commonly experienced symptoms were vaginal discharge (83%), followed by vulval itching (78.5%), lower abdominal pain (75.8%), backache (65.7%), urinary tract infections (24.4%), perianal pain (20.6%), dysuria (17.9%), polyuria (14.6%), and abnormal vaginal growth (4.8%). In contrast, the most prevalent symptoms among low-risk RTI participants were lower abdominal pain (58.5%) and backache (58.1%).

#### 3.2.2 Gynecological diagnostic history of participants belonging to low- and high-risk RTI

The gynecological diagnostic history of participants from both low- and high-risk RTI groups was examined. Participants were asked whether they had ever been diagnosed with an RTI. Among the total participants (*N* = 1,241), approximately 5% (*N* = 57) had a documented history of gynecological diagnosis.

The most commonly diagnosed gynecological condition was pelvic inflammatory disease (affecting the endometrium, fallopian tubes, and ovaries), accounting for 42.9% and 39.5% in low-risk and high-risk RTIs respectively (Supplementary Figure S1). However, vaginitis was notably more prevalent in the high-risk RTI group (37.2%) compared to the low-risk group (28.6%).

Other reproductive infections, such as vulval and Bartholin gland infections, were slightly more frequent in the low-risk RTI group (21.4%) than in the high-risk RTI group (16.3%). Cervicitis was the least reported RTI, with similar rates in both groups (7.0% and 7.1%, respectively; Supplementary Figure S1).

### 3.3 Association of sociodemographic, reproductive health, hygiene, healthcare literacy, and vaccination status with RTI risk levels

#### 3.3.1 Sociodemographic factors

In this study, sociodemographic variables were not found to be associated with low- or high-risk RTI (Table 2). The majority of participants (over 93%) across all three groups were between 18 and 25 years old. Most participants had a normal weight, were unmarried, and had at least a twelfth-grade education.

**Table 2:** Association of demographic factors with symptoms of reproductive tract infections in urban females.

Variables	RTI absent *N* (%) 679 (35.4)	Low-risk RTI *N* (%) 446 (23.2)	High-risk RTI *N* (%) 795 (41.4)	Low-risk RTI vs. RTI absent *χ*^2^ *P*-value	High-risk RTI vs. RTI absent *χ*^2^ *P*-value	Low-risk RTI vs. high-risk RTI *χ*^2^ *P*-value
Age (years)						
18–25	628 (93.7)	418 (94.8)	742 (95.2)	4.840	4.413	8.988
26–50	42 (6.3)	23 (5.2)	37 (4.8)	0.184	0.220	0.029*
BMI status						
Underweight (<18.5 Kg/m^2^)	143 (23.0)	90 (22.3)	154 (20.7)	0.210	2.119	2.119
Normal weight (18.5–24 Kg/m^2^)	386 (62.2)	253 (62.6)	465 (62.4)	0.976	0.548	0.548
Overweight (25–29.9 Kg/m^2^)	67 (10.8)	46 (11.4)	104 (14.0)			
Obese (>30 Kg/m^2^)	25 (4.0)	15 (3.7)	22 (2.9)			
Marital status						
Unmarried youth	651 (95.9)	426 (95.5)	767 (96.5)	0.932	1.570	3.144
Married	28 (4.1)	20 (4.5)	28 (3.5)	0.818	0.666	0.370
Education status						
XII grade	461 (67.9)	307 (68.8)	574 (72.2)	2.348	3.449	5.318
Graduate	179 (26.4)	105 (23.5)	185 (23.3)	0.309	0.178	0.070
Postgraduate and above	39 (5.7)	34 (7.6)	36 (4.5)			
Occupation						
Students	626 (92.2)	408 (91.5)	742 (93.3)	1.858	3.884	2.894
Academician/Government/Private/Healthcare workers	38 (5.6)	29 (6.5)	43 (5.4)	0.762	0.422	0.576
Home makers	15 (2.2)	9 (2.0)	10 (1.3)			

* 98.33% Confidence intervals were calculated after correction to significance level using Bonferroni method.

#### 3.3.2 Reproductive, menstrual hygiene, and healthcare literacy factors

The age of menarche onset was significantly different between females in the high-risk RTI and RTI-absent groups. A majority (72.7%) of females in the RTI-absent group reported menarche onset between the ages of 12 and 16, compared to those in the high-risk RTI group.

Additionally, females in the high-risk RTI group differed significantly from those in the RTI-absent group regarding previously diagnosed reproductive tract diseases and RTI/STI (Table 3). Females in both the low- (35.0%) and high-risk (30.3%) RTI groups also showed significant differences from the RTI-absent group (4.9%) regarding prior use of medication for RTI symptoms within the last three months.

**Table 3:** Association between menstrual health, contraceptive awareness, and symptoms suggestive of reproductive tract infection in urban females.

Variables	RTI absent *N* (%) 679 (35.4)	Low-risk RTI *N* (%) 446 (23.2)	High-risk RTI *N* (%) 795 (41.4)	Low-risk RTI vs. RTI absent *χ*^2^ *P*-value	High-risk RTI vs. RTI absent *χ*^2^ *P*-value	Low-risk RTI vs. high-risk RTI*χ*^2^ *P*-value
Menarche onset (Years)						
<10	5 (0.8)	6 (1.4)	8 (1.0)	11.230	16.080	0.351
10–12	144 (22.8)	135 (30.5)	244 (30.9)	0.011*	0.001*	0.951
12–16	459 (72.7)	294 (66.4)	525 (66.5)			
>16	23 (3.7)	8 (1.8)	13 (1.6)			
Menstrual cycle status						
Irregular	94 (15.2)	50 (11.4)	157 (19.9)	2.706	5.053	13.805
Regular	526 (84.8)	387 (88.6)	631 (80.1)	0.100*	0.025*	0.002**
Length (days)						
>21 to <35	481 (79.5)	346 (84.0)	580 (75.6)	3.374	3.682	11.201
>35 to <45	97 (16.0)	50 (12.1)	138 (18.0)	0.185	0.159	0.004**
>45	27 (4.5)	16 (3.9)	49 (6.4)			
Previously diagnosed with reproductive diseases						
Yes	11 (1.6)	14 (3.1)	43 (5.4)	2.202	11.759	2.025
No	668 (98.4)	432 (96.9)	752 (94.6)	0.138	0.001**	0.155
Previously diagnosed with RTIs/STIs						
Yes	1 (0.1)	3 (0.7)	19 (2.4)	0.873	12.116	3.903
No	677 (99.9)	443 (99.3)	776 (97.6)	0.350	0.001**	0.048
Use of medication for RTI/UTI in the past 3 months						
Yes	33 (4.9)	156 (35.0)	241 (30.3)	172.231	154.822	2.645
No	645 (95.1)	290 (65.0)	554 (69.7)	0.000**	0.000**	0.104
Menstrual hygiene practices						
Yes	640 (94.4)	439 (98.4)	789 (99.2)	10.372	28.053	1.128
No	38 (5.6)	7 (1.6)	6 (0.8)	0.001**	0.000**	0.288
Awareness of contraceptive methods						
Yes	404 (70.9)	297 (78.8)	574 (84.7)	6.965	33.893	5.418
No	166 (29.1)	80 (21.2)	104 (15.3)	0.008**	0.000**	0.020**

* 98.33% Confidence intervals were calculated after correction to significance level using Bonferroni method.

Regarding menstrual hygiene practices, the majority of females (>94%) reported maintaining menstrual hygiene practices. However, adherence was significantly higher among females in the low- and high-risk RTI groups than in the RTI-absent group (Table 3). Similarly, healthcare literacy about contraceptive methods was significantly greater (>78%) among females in the RTI-present groups compared to the RTI-absent group.

#### 3.3.3 History of co-morbidities

When comparing comorbidities, a significantly higher percentage of participants in the high-risk RTI group (68.1%) reported having family members who had suffered or were suffering from a comorbidity, compared to those in the low-risk (58.5%) and RTI-absent (51.8%) groups (Supplementary Table S1). However, the percentage of participants with medical conditions was similar across all three groups, ranging from 22.4% to 25.7%.

Among the comorbidities reported in family members or relatives, diabetes was the most common, followed by hypertension, cancer, cardiovascular diseases,

PCOS, endometriosis, and thyroid disorders (Supplementary Figure S2). Asthma was the least reported comorbidity and was observed only in low and high-risk RTI groups.

**Figure 3:**
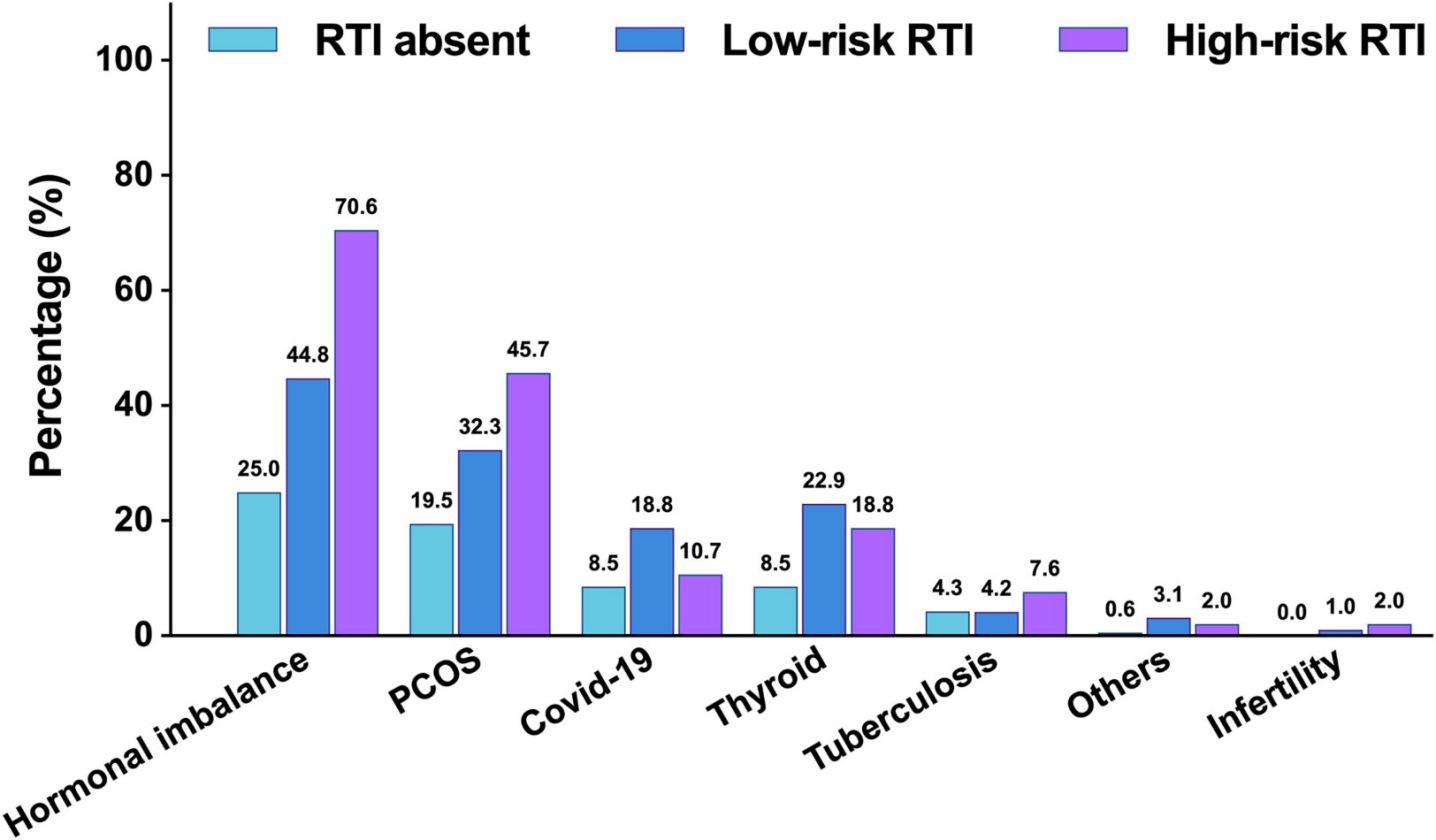
Distribution of medical conditions Among RTI-absent, Low-risk RTI, and high-risk RTI participants. This bar chart presents the prevalence of various medical conditions among participants categorized into RTI-absent, low-risk RTI, and high-risk RTI groups. The *x*-axis represents different medical conditions, while the *y*-axis denotes the percentage of participants affected. RTI, Reproductive Tract Infections; PCOS, Polycystic Ovarian Syndrome. The percentage for each disease is displayed above its corresponding bar in the graph.

Similarly, when comparing medical conditions among participants, hormonal imbalances and PCOS were the most prevalent across all groups (Figure 3). However, the prevalence of these conditions was significantly higher in the high-risk RTI group (70.6% and 45.7%, respectively) than in the low-risk RTI (44.8% and 32.3%) and RTI-absent (25% and 19.5%) groups. Other reported medical conditions included COVID−19, thyroid disorders, and tuberculosis across all three groups. Additionally, 5.7% of participants reported other conditions such as skin problems, hypertension, and asthma. Infertility was the least reported medical condition and was found only among participants in the low- and high-risk RTI groups.

#### 3.3.4 Vaccines against RTI

The HPV vaccine uptake was higher in the low-risk RTI group compared to the high-risk and RTI-absent groups (Supplementary Table S2). Additionally, hepatitis B vaccination rates were similar across all three groups.

### 3.4 Multinomial logistic regression analysis

Multinomial logistic regression analysis was conducted to identify the risk factors associated with low- and high-risk RTI (Tables 4, 5).

**Table 4:** A multinomial logistic regression analysis of factors associated with Low-risk of reproductive tract infections.

Variables	OR	95% CI	*P*-value
Age (Years)	1.037	[0.799–1.345]	0.785
BMI	0.892	[0.654–1.217]	0.468
Marital status	0.619	[0.372–1.027]	0.064
Highest level of education	0.943	[0.726–1.224]	0.659
Occupation	1.123	[0.831–1.520]	0.445
Prescription medication usage in the last 3 months for STI/RTI symptoms	10.642	[6.659–17.016]	<0.001*
Age at menarche (First Period)	0.572	[0.442–0.740]	<0.001*
Menstrual cycle description	1.365	[0.854–2.181]	0.194
Length of menstrual cycle	0.815	[0.589–1.127]	0.216
Family history of medical conditions	0.968	[0.726–1.292]	0.826
Presence of chronic illness	0.803	[0.573–1.125]	0.202
History of symptoms related to RTI/STI	0.987	[0.618–1.577]	0.956
Previously diagnosed of STI/RTIs	1.749	[0.669–4.574]	0.254
Menstrual hygiene practices	1.368	[0.703–2.661]	0.356
Awareness of contraceptive methods	1.475	[1.024–2.125]	0.037*
HPV vaccination status	0.707	[0.423–1.186]	0.189
Hepatitis B vaccination status	0.823	[0.205–0.447]	0.307

BMI, body mass index; OR, odds ratio; CI, confidence interval; RTI, reproductive tract infection; STI, sexually transmitted infection; *P < 0.05. Reference category: RTI-absent group (*N* = 679). All variables were entered simultaneously (Enter method). Model fit: LR *χ*^2^(17) = 251.4, *p* < 0.001; Nagelkerke pseudo-R^2^ = 0.178; Cox & Snell pseudo-R^2^ = 0.122; *N* = 1,920. VIF values for all factors <2.3 (see Supplementary Table S3).

**Table 5:** A multinomial logistic regression analysis of factors associated with high-risk of reproductive tract infections (RTIs).

Variables	OR	95% CI	*P*-value
Age (Years)	1.128	[0.898–1.418]	0.299
BMI	1.100	[0.532–1.131]	0.321
Marital status	0.516	[0.320–0.834]	0.007*
Highest level of education	0.796	[0.628–1.010]	0.060
Occupation	1.247	[0.962–1.615]	0.095
Prescription medication usage in the last 3 months for STI/RTI symptoms	8.839	[5.631–13.878]	<0.001*
Age at menarche (First Period)	0.606	[0.481–0.764]	<0.001*
Menstrual cycle description	0.718	[0.491–1.048]	0.086
Length of menstrual cycle	1.042	[0.804–1.351]	0.754
Family history of medical conditions	1.494	[1.158–1.927]	0.002*
Presence of chronic illness	1.008	[0.758–1.340]	0.958
History of symptoms related to RTI/STI	0.839	[0.561–1.253]	0.390
Previously diagnosed of STI/RTIs	1.406	[0.581–3.404]	0.450
Menstrual hygiene practices	3.183	[1.668–6.072]	<0.001*
Awareness of contraceptive methods	1.919	[1.380–2.669]	<0.001*
HPV vaccination status	0.878	[0.573–1.345]	0.549
Hepatitis B vaccination status	0.721	[0.484–1.075]	0.108

BMI, body mass index; OR, odds ratio; CI, confidence interval; RTI, reproductive tract infection; STI, sexually transmitted infection; *P < 0.05. Reference category: RTI-absent group (*N* = 679). All variables entered simultaneously (Enter method). Model fit: LR *χ*^2^(17) = 338.6, *p* < 0.001; Nagelkerke pseudo-R^2^ = 0.221; Cox & Snell pseudo-R^2^ = 0.161; *N* = 1,920. VIF values for all factors <2.3 (see Supplementary Table S3).

#### 3.4.1 Significant predictors in the low-risk RTI group

The analysis of the low-risk RTI group identified several significant predictors (Table 4). Prior medication usage (*P* < 0.001) and age at menarche (*P* < 0.001) showed significant associations. Additionally, healthcare literacy about contraceptive methods (*P* = 0.037) was found to be a significant predictor for low-risk RTI.

#### 3.4.2 Significant predictors in the high-risk RTI group

The analysis of the high-risk RTI group revealed a distinct pattern of significant predictors (Table 5). Marital status showed a significant association (OR = 0.516, *P* = 0.007). Family history of medical conditions played a critical role (OR = 1.494, *P* = 0.002). Prior RTI/STI medication use in the last 3 months was the strongest predictor (OR = 8.839, *P* < 0.001), indicating a high burden of active or recent infection in this group. Age at menarche was also a significant predictor (OR = 0.606, *P* < 0.001). Additionally, menstrual hygiene practices (OR = 3.183, *P* < 0.001) and contraceptive method awareness (OR = 1.919, *P* < 0.001) were associated with high-risk RTI.

### 3.5 Perception towards emerging RTI/STI vaccines

Participants were asked, “If available today, would you be interested in receiving a vaccine to prevent RTIs/STIs?” Among the 1,920 participants, 46.2% (*N* = 888) expressed willingness to receive an RTIs/STIs vaccine, indicating vaccine acceptance, while 53.8% (*N* = 1,032) were not interested, reflecting vaccine hesitancy.

**Figure 4:**
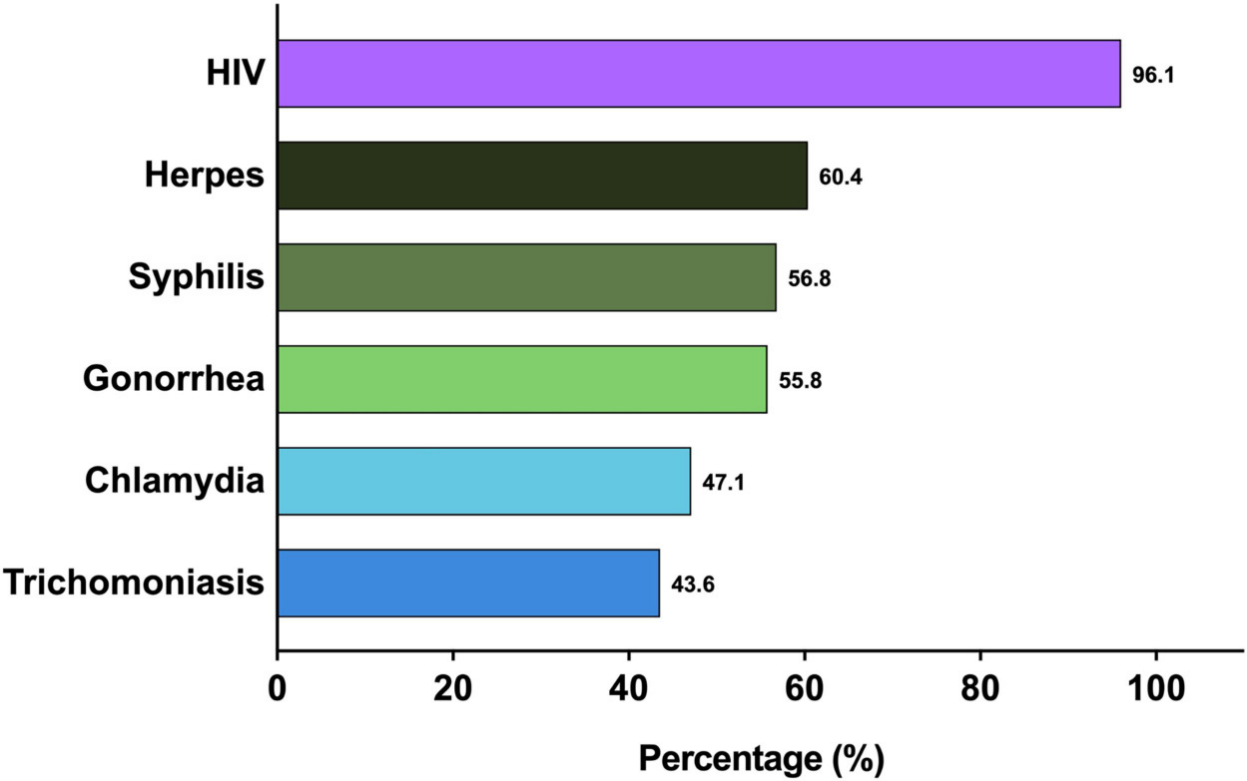
Distribution of diseases for which participants expressed interest in vaccination. This horizontal bar chart presents the percentage of participants interested in receiving vaccines for sexually transmitted infections (STIs). The *x*-axis represents the percentage of participants, while the *y*-axis lists the specific diseases for which vaccination interest was recorded.

When asked, “If and when available, which of the following STI vaccines would you be interested in receiving?”, 56.8% (*N* = 508) of vaccine-accepting participants responded. Among them, 96.1% showed interest in an HIV vaccine, followed by herpes (60.4%), syphilis (56.8%), gonorrhea (55.8%), chlamydia (47.1%), and trichomoniasis (43.6%) (Figure 4).

Additionally, participants were asked, “If STI vaccines were available, when do you think it would be best to first offer STI vaccines?” The majority (48.3%) believed adolescence was the ideal time, followed by early adulthood (23.1%).

### 3.6 Factors influencing RTI/STI vaccine perception among urban females

#### 3.6.1 Socio-demographic factors

Participants were categorized into two groups based on their intention to receive an RTI/STI vaccine: the vaccine acceptance group and the vaccine-hesitant group (Table 6). The Chi-square test (*χ*^2^) was used to assess demographic differences between these groups.

**Table 6:** Comparison of demographic, health, and awareness factors between vaccine acceptance (*N* = 888, 46.2%) and Hesitancy Groups (*N* = 1,032, 53.8%).

Category	Characteristics	Vaccine acceptance (*N*, %)	Vaccine hesitance (*N*, %)	*χ* ^2^	*P*-value
Demographic Factors	Age (Years)				
	18–25	831 (94.8)	957 (94.5)	0.074	0.786
	26–50	46 (5.2)	56 (5.5)		
	Life Stage				
	Unmarried youth	850 (96.8)	994 (96.3)	0.346	0.556
	Married	28 (3.2)	38 (3.7)		
Health-Related Factors	BMI				
	Underweight (<18.5 Kg/m^2^)	148 (18.2)	239 (25.0)	57.623	<0.001**
	Normal weight (18.5–24 Kg/m^2^)	484 (59.5)	620 (64.9)		
	Overweight (25–29.9 Kg/m^2^)	149 (18.3)	68 (7.1)		
	Obese (>30 Kg/m^2^)	33 (4.0)	29 (3.0)		
	Occurrence of reproductive tract disease (Past 3 Months)				
	Yes	27 (3.0)	38 (3.7)	0.420	0.516
	No	861 (97.0)	994 (96.3)		
	Suffering from chronic conditions				
	Yes	217 (25.3)	240 (24.1)	0.304	0.581
	No	642 (74.7)	758 (75.9)		
Awareness and knowledge factors	Understanding about STI				
	Poor	91 (13.5)	117 (15.4)	1.227	0.541
	Moderate	387 (57.5)	420 (55.1)		
	Good	195 (29.0)	225 (29.5)		
	HPV vaccine awareness				
	Yes	484 (54.6)	558 (54.1)	0.029	0.862
	No	403 (45.4)	474 (45.9)		
	Hepatitis B vaccine awareness				
	Yes	702 (80.4)	796 (78.8)	0.642	0.423
	No	171 (19.6)	214 (21.2)		
Vaccination status	HPV vaccine				
	Yes	76 (8.6)	82 (7.9)	0.244	0.620
	No	811 (91.4)	950 (92.1)		
	Hepatitis B vaccine				
	Yes	242 (27.3)	255 (24.7)	1.646	0.199
	No	645 (72.7)	777 (75.3)		
Perceptions and attitudes towards STI vaccines	Preferred age for STI vaccine introduction				
	Infancy	41 (4.6)	47 (4.6)	2.990	0.560
	Childhood	90 (10.2)	119 (11.5)		
	Adolescence	446 (50.3)	481 (46.7)		
	Early Adulthood	196 (22.1)	248 (24.0)		
	Adulthood/Late Adulthood	114 (12.8)	136 (13.2)		
	Financial willingness to pay for vaccination				
	Yes	260 (29.3)	305 (29.5)	0.029	0.985
	Maybe	418 (47.1)	487 (47.2)		
	No	209 (23.6)	240 (23.3)		
	Encouraging partner(s) to get immunized				
	Agree	643 (72.5)	736 (71.3)	0.328	0.849
	Neutral	218 (24.6)	264 (25.6)		
	Disagree	26 (2.9)	32 (3.1)		
	Belief in the effectiveness of vaccination in preventing STIs				
	Agree	587 (66.2)	671 (65.0)	0.860	0.650
	Neutral	276 (31.1)	326 (31.6)		
	Disagree	24 (2.7)	35 (3.4)		

BMI, body mass index; HPV, human papillomavirus; STI, sexually transmitted infection.

Most characteristics, including age, life stage, RTI occurrence, and chronic conditions, were similar between the two groups. However, BMI (*χ*^2^ = 57.62, *P* < 0.001) showed a statistically significant difference. The percentage of underweight participants was higher in the vaccine-hesitant group (25.0%) compared to the vaccine-acceptance group (18.2%).

#### 3.6.2 Healthcare literacy among vaccine-accepting and vaccine-hesitant participants

Participants were asked about their understanding of STIs, and no significant differences were observed between the vaccine-acceptance and vaccine-hesitant groups. However, the majority in both groups demonstrated moderate knowledge (57.5% vs. 55.1%) (Table 6). Additionally, participants were assessed on their awareness of STI vaccines, specifically HPV and hepatitis B. The results indicated that both groups had nearly equal levels of awareness about these vaccines (Table 6). However, a significantly higher percentage of participants (∼80%) were aware of the hepatitis B vaccine, compared to ∼54.6% for the HPV vaccine.

Similarly, the percentage of participants who had received the hepatitis B vaccine (27.3%) was notably higher than those who had received the HPV vaccine (8.6%). However, no significant differences were observed in the distribution of HPV and hepatitis B vaccination rates between the vaccine acceptance and vaccine-hesitant groups.

**Figure 5:**
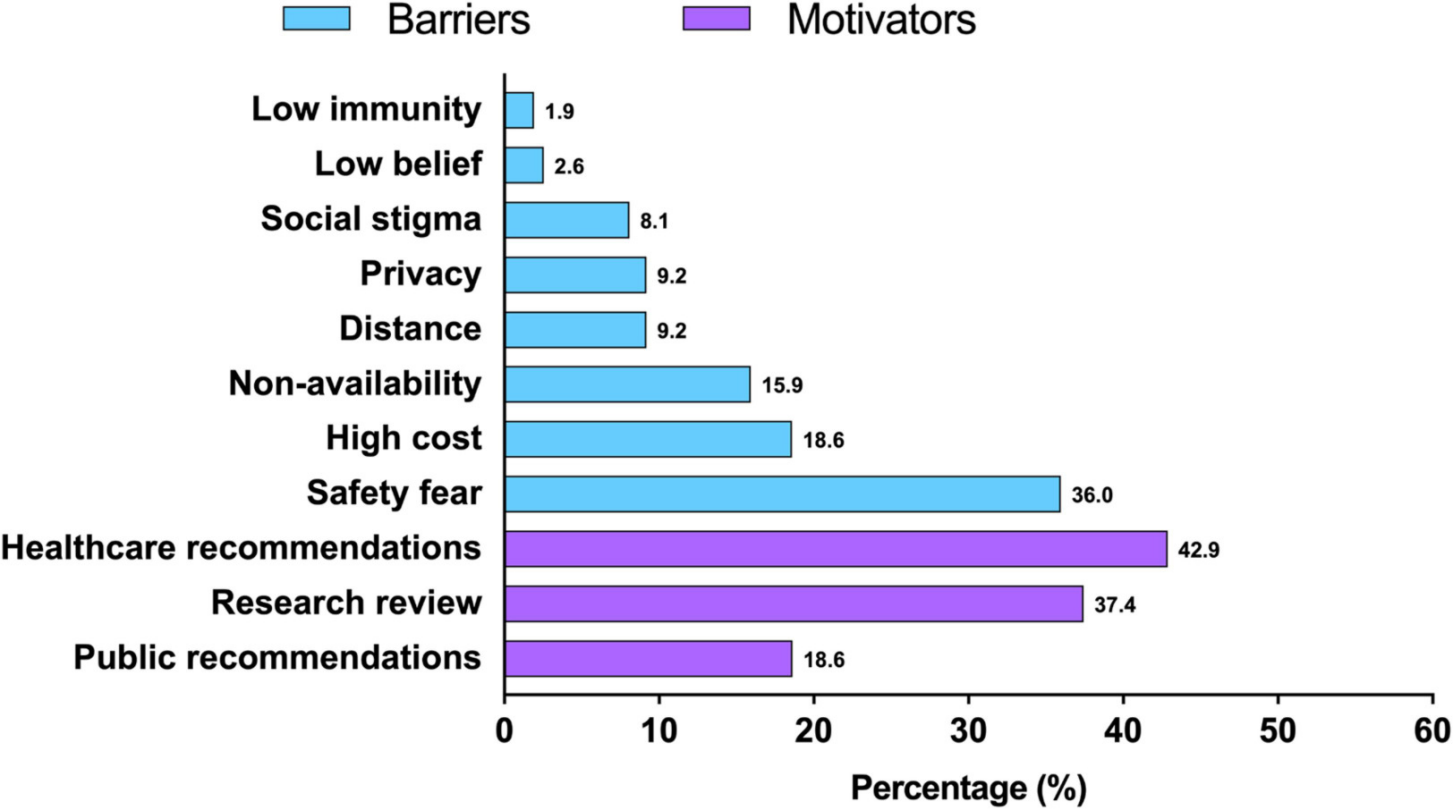
Barriers and motivators shaping interest in sexually transmitted infection (STI) vaccination. This figure presents the distribution of barriers (blue) and motivating factors (purple) associated with individuals’ reluctance or willingness to receive an STI vaccine. The *x*-axis represents the percentage of respondents reporting each factor. The percentage of each source is displayed above its corresponding bar in the graph.

Participants were also asked, “Do you think STI vaccination could be an effective way to prevent STIs?” No significant differences were observed between the vaccine acceptance and vaccine-hesitant groups regarding their belief in vaccine effectiveness. Notably, even among those hesitant to receive the vaccine, 65% still believed in its effectiveness. Given this finding, we further examined barriers to STI vaccination (Figure 5).

#### 3.6.3 Predictors of vaccine acceptance: binary logistic regression analysis

To identify independent predictors of STI/RTI vaccine acceptance, a binary logistic regression analysis was performed (*N* = 1920 with complete covariate data; reference category: vaccine-hesitant group). The overall model was statistically significant: LR *χ*^2^(7) = 169.79, *P* < 0.001; Nagelkerke pseudo-*R*^2^ = 0.131; McFadden pseudo-R^2^ = 0.079, indicating that the included predictors collectively explained meaningful variance in vaccine acceptance. Results are presented in Table 7.

**Table 7:** A binary logistic regression analysis of predictors of STI/RTI vaccine acceptance among urban females (*N* = 1920).

Variables	OR	95% CI	*P*-value
Age (Years)	0.672	[0.328–1.379]	0.279
Presence of RTI symptoms	1.947	[1.376–2.756]	0.001*
Marital status	0.342	[0.148–0.789]	0.012*
Understanding about STI	1.264	[1.035–1.543]	0.021*
Awareness of HPV vaccine	2.262	[1.790–2.860]	0.001*
Awareness of Hepatitis B Vaccine	1.762	[1.367–2.273]	<0.001*
Presence of chronic illness	0.978	[0.764–1.253]	0.860

OR, odds ratio; CI, confidence interval; Sig., statistical significance (*p < 0.05; not significant). Reference category: vaccine-hesitant (*N*o). Model: LR *χ*^2^(7) = 169.79, *p* < 0.001; Nagelkerke R^2^ = 0.131.

HPV vaccine awareness [OR = 2.262, 95% CI (1.790–2.860), *P* < 0.001] and hepatitis B vaccine awareness [OR = 1.762, 95% CI (1.367–2.273), *P* < 0.001] were the strongest predictors of acceptance, suggesting that familiarity with existing STI vaccines substantially increases willingness to accept emerging ones. RTI symptom presence was also a significant predictor [OR = 1.947, 95% CI (1.376–2.756), *P* = 0.001], indicating that personal experience with RTI symptoms motivates vaccine acceptance. Higher STI knowledge was independently associated with greater acceptance [OR = 1.264, 95% CI (1.035–1.543), *P* = 0.021]. Married participants were significantly less likely to accept vaccines compared to unmarried participants [OR = 0.342, 95% CI (0.148–0.789), *P* = 0.012], potentially reflecting partner dynamics, perceived risk, or social stigma. Age and chronic illness status were not significant predictors in this model.

#### 3.6.4 Barriers to STI vaccine acceptance

Participants reported various barriers to receiving an STI vaccine (Figure 5). The most common concern was vaccine safety (36%), followed by cost concerns (18.6%).

Other reported barriers included vaccine non-availability (15.9%) and the inconvenience of accessing a clinic due to distance (9.2%). Additionally, privacy concerns were a factor, with 9.2% of participants worried about having STI vaccination recorded in their health records and 8.1% citing social stigma as a deterrent.

The least prevalent barriers were trust-related concerns, such as low belief in vaccines (2.6%) and health-related concerns including low immunity, older age, or comorbidities (1.9%).

#### 3.6.5 Motivating factors for STI vaccine acceptance among the hesitant group

Among participants who answered the question, “Which of the following would be helpful in accepting an STI vaccine?”, the most common motivating factor was a recommendation from a doctor or healthcare provider (∼43%) (Figure 5).

Additionally, 37.4% of participants stated they would be motivated after reviewing detailed research on vaccine adverse events and their constituents. A smaller yet notable proportion (18.6%) expressed concerns about vaccine safety, indicating they would wait to observe others’ reactions before making a decision.

#### 3.6.6 Preferred and trusted sources of information on STI vaccines

**Figure 6:**
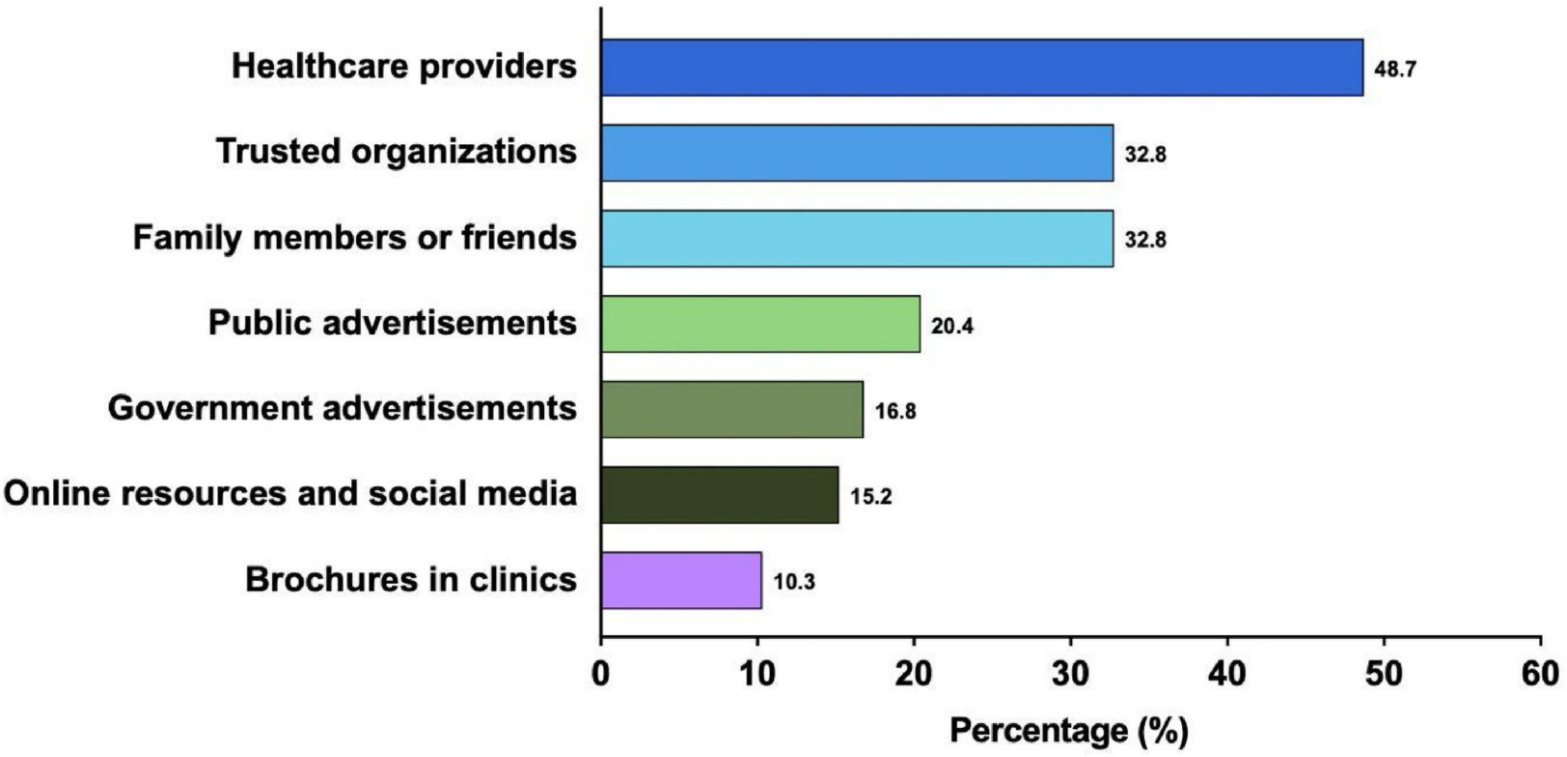
Trusted sources of information about sexually transmitted infection (STI) vaccination. This figure illustrates the distribution of trusted sources from which individuals seek information regarding STI vaccines. The *x*-axis represents the percentage of respondents who consider each source reliable.

Participants were asked, “Where would you like to receive information about STI vaccines?” As shown in Figure 6, the most trusted source was healthcare providers (48.7%), followed by reliable organizations (32.8%), such as Centers for Disease Control and Prevention (CDC), and WHO.

Other sources included family members and friends (32.8%), public advertisements (20.4%), and government advertisements (16.8%). Additionally, 15.2% of participants reported relying on online resources and social media, while 10.3% obtained information from brochures in clinics.

## 4 Discussion

This study aimed to determine the prevalence and risk factors of reproductive tract infections (RTIs) among urban females. The findings reveal a substantial burden of RTI symptoms, with ∼65% of participants overall and ∼41% classified as high-risk. These symptoms are commonly linked to RTI such as bacterial vaginosis and candidiasis. Abnormal vaginal discharge was the most frequently reported symptom, aligning with previous studies (25, 26). Additionally, abdominal pain and backache, experienced by more than 65% of participants, were consistent with prior findings among North Indian females (12, 27). Despite the high prevalence of symptoms, only 5% of participants reported a gynecological diagnosis of RTI/STI, suggesting significant underdiagnosis and underreporting. Factors contributing to this discrepancy may include social stigma, lack of awareness, and limited access to healthcare services. Pelvic inflammatory disease (42.9% and 39.5%) was the most common gynecological condition in both low- and high-risk RTI groups, while vaginitis (37.2%) was more prevalent in the high-risk RTI group. These findings align with previous research (28) and underscore the importance of regular screening and comprehensive treatment plans to effectively manage RTI.

Interestingly, unlike previous studies that identified age, marital status, and education as critical socio-demographic determinants of RTI risk (28, 29), the present study found no significant association with these variables. This lack of variability could be attributed to the predominant participation of urban females, who often share more homogeneous socio-demographic profiles, including similar levels of education and marital patterns, which may have influenced the findings.

For the first time, this study identified a significant association between family comorbidities, particularly diabetes and hypertension in high-risk RTI. The observed association between familial comorbidity and RTI risk in our study is a novel finding, as no previous literature directly links these variables. These preliminary observations highlight an avenue for future longitudinal research to better understand possible shared familial or household determinants of RTI risk. A possible reason could be that the genetic predisposition to diabetes may lead to impaired immune response, increasing susceptibility to infections. These observations emphasize the importance of addressing comorbid conditions in reproductive health management. Menstrual health factors also play a crucial role in RTI risk. According to previous studies, the variations in menstrual cycle length had a significantly higher likelihood of high-risk RTI (30, 31). Additionally, hormonal imbalances, particularly PCOS, were more prevalent in the high-risk RTI group, reinforcing the role of hormonally mediated immune responses in increasing susceptibility to infections. These results align with previous research PCOS to RTI (32, 33). Regular monitoring and timely PCOS management could help mitigate RTI risk. Another potential risk factor was early menarche. Females who experienced menarche between ages 10–12 were more likely to report both low- and high-risk RTI compared to those with menarche onset at 12–16 years or older. Early menarche is a known risk factor for reproductive health issues, likely due to prolonged estrogen exposure and early sexual initiation (34, 35). The association between menstrual hygiene practices and higher RTI symptom risk (OR = 3.183) must be interpreted with caution given the cross-sectional design. Only 5.6% of participants (*N* = 38) reported no hygiene practices, constituting a very small reference group, the odds ratio therefore compares the overwhelming majority of participants against an atypically small, potentially poorly characterized reference group, limiting the reliability of this estimate. More importantly, reverse causality cannot be excluded in this cross-sectional design: women with RTI symptoms may intensify hygiene practices in response to discomfort, or may be more attentive to and likely to report hygiene behaviors when symptomatic. Consistent with this interpretation, sensitivity analyses excluding participants who had used RTI/UTI medication in the past three months showed substantial attenuation of the hygiene OR [OR = 2.21, 95% CI (0.96–5.09), *P* = 0.063], with further attenuation after additionally excluding those with a prior RTI diagnosis (OR = 1.87, *P* = 0.203, Supplementary table S4). These findings suggest that the hygiene association in the main analysis is largely driven by symptomatic or previously infected women and should not be interpreted as evidence that menstrual hygiene increases RTI risk. Similarly, the association between contraceptive awareness and RTI risk likely reflects greater healthcare engagement among symptomatic women rather than a causal pathway, the sensitivity analyses showed this association to be more stable, consistent with higher health-seeking behavior in the RTI-present group. Notably, this study did not capture data on actual contraceptive use due to the sensitive nature of this information, so findings reflect awareness rather than usage patterns (36). Longitudinal studies with objective hygiene assessment are needed to clarify these relationships.

A history of RTIs was significantly associated with high-risk RTI in this study, suggesting that recurrent or untreated infections can contribute to progressive deterioration of reproductive health. Females with a prior history of RTIs are likely to experience repeated episodes due to incomplete treatment, antimicrobial resistance, or misdiagnosis. A considerable proportion of participants (35.0% in the low-risk and 30.3% in the high-risk groups) reported having taken medication for RTIs/UTIs symptoms within the past three months, highlighting the ongoing burden of symptomatic infections even among low-risk females. These findings, in line with previous studies reporting recurrent RTIs in prior patients (12, 37), indicate that past infections are a strong predictor of current high-risk status and emphasize the need for timely diagnosis, appropriate therapy, and follow-up to break the cycle of reinfection.

In addition to RTI prevalence and risk factors, this study examined urban females’ willingness to receive STIs/RTIs vaccinations. Consistent with prior research, low uptake of HPV and hepatitis B vaccines remains a significant public health challenge, particularly in low- and middle-income countries (38). While most HPV vaccinations occurred during adolescence (ages 11–18), in line with global immunization guidelines, hepatitis B vaccinations were primarily received during infancy, reflecting successful early immunization programs (39–41). However, as vaccination data were self-reported, there is a possibility of recall bias, especially for vaccines administered in early childhood, which could affect the accuracy of reporting. In addition, vaccination efforts for older populations remain inadequate, highlighting the need for targeted awareness campaigns. Logistic regression analysis identified several significant predictors of vaccine acceptance beyond descriptive patterns. Awareness of existing STI vaccines- particularly HPV and hepatitis B was the strongest predictor of willingness to accept emerging STI vaccines, suggesting a vaccine familiarity spillover effect, those already familiar with established STI vaccines are substantially more open to new ones. This underscores the value of HPV and hepatitis B vaccination programs not only for their direct disease-prevention benefits, but also as a gateway to broader STI vaccine literacy. RTI symptoms experience was also a significant predictor, implying that personal health burden motivates preventive behavior-a finding consistent with the health belief model and prior vaccine acceptance literature (42, 43). Greater STI knowledge independently predicted acceptance, reinforcing the critical role of health education programs. Notably, married women showed significantly lower acceptance odds, which may reflect greater social stigma around STI-related vaccination, reduced perception of personal risk, or partner and family influences on health decisions in this population. These findings provide actionable targets for public health intervention, vaccine awareness campaigns linked to existing HPV and hepatitis B programs, tailored messaging for married women, and community health literacy initiatives are likely to be the most effective levers for improving STI/RTI vaccine acceptance in this population.

This study found a moderate vaccine acceptance rate (46.2%), with a high preference for vaccines against HIV, herpes, and syphilis, indicating awareness of infection severity and a willingness to seek protection. The preference for adolescent vaccination (50.3%) aligns with WHO recommendations for early immunization (39, 44). While overall knowledge about HPV and Hepatitis B vaccine was similar between the vaccine-accepting and vaccine-hesitant groups, a higher percentage of participants were aware of the hepatitis B vaccine compared to HPV, suggesting that targeted educational efforts could improve HPV vaccine uptake, which remains below average despite its proven efficacy (45). Vaccine cost was a significant determinant of emerging STI vaccines acceptance, indicating that financial barriers contribute to vaccine hesitancy, particularly in developing countries (46). Due to the sensitivity of the topic, the study did not directly question respondents about their belief in partner transmission. However, the finding that more than 50% of participants were willing to encourage their partners to get immunized suggests an implicit understanding of the potential role of partners in disease prevention. It highlights a broader community health benefit. Although vaccine hesitancy was prevalent, 65% of participants believed in vaccine efficacy, suggesting that factors beyond efficacy-such as cost and accessibility-contribute to hesitancy. Vaccine safety concerns were the most cited barrier, followed by cost, availability, and access (47). Social stigma surrounding STI vaccines also emerged as a major issue, emphasizing the need for sensitization efforts to improve acceptance (48).

Interestingly, attitudinal barriers such as low belief in vaccines and immunity concerns were less common, suggesting that misconceptions about vaccine effectiveness are not the main drivers of hesitancy. The strongest motivator for vaccine acceptance was a recommendation from a healthcare provider, followed by a detailed research review, reinforcing previous findings that healthcare professionals play a crucial role in addressing vaccine concerns (42, 43). Similarly, healthcare providers were the most trusted source of information, followed by WHO, CDC, family and friends, and public advertisements (16, 49).

## 5 Conclusion

This study highlights a high prevalence of symptomatic RTIs among urban females, with key contributing factors including family history of comorbidities, early menarche, irregular menstrual cycles, and inadequate menstrual hygiene. Vaccine hesitancy, primarily driven by safety and cost concerns, underscores the need for enhanced awareness, improved access, and stronger preventive healthcare measures.

Given the established link between RTI and increased pelvic inflammatory diseases, infertility, and morbidity, these findings further reinforce the urgent need for integrated screening and awareness programs, expanding HPV and Hepatitis B vaccine coverage, and addressing social stigma. This could significantly reduce the burden of RTIs, particularly in resource-limited settings. Additionally, future vaccine development efforts, including emerging multi-pathogen STI vaccines, should consider these findings to enhance public acceptance and uptake. Strengthening healthcare infrastructure, provider recommendations, and targeted awareness campaigns remains essential to mitigate RTI/STI-associated risks and reduce overall infection rates.

## 6 Limitations of the study

This study did not include clinical confirmation and laboratory tests for diagnosing RTI/STIs and instead relied on a syndromic approach, which may not accurately estimate the true prevalence of infections in the community. Additionally, the syndromic approach may have excluded asymptomatic RTI cases, potentially leading to an underestimation of the actual disease burden.

Social stigma surrounding RTI may have also discouraged some females from reporting their symptoms, further contributing to underreported prevalence rates. Another limitation is the absence of data on sexual health behaviors, which are important determinants of RTI risk but were not included in this study due to the sensitive nature of such questions. The limitation of the study also includes age distribution of the sample, with a heavy representation (over 90%) from the 18–25 age group. Although a small percentage of participants from other age groups were included to broaden the community perspective, the findings may not be fully generalizable to older or non-student populations. Lastly, this study did not include follow-up with participants who sought or were encouraged to seek treatment, limiting insights into treatment adherence and long-term health outcomes.

## Data Availability

The datasets will be available on request from the corresponding authors.
