# Supplementary material for: Prevalence, risk factors, and perceptions of vaccination against reproductive tract infections among urban females in Delhi: a cross-sectional study
Source: Front Reprod Health. 2026 May 26;8:1812966. doi: 10.3389/frph.2026.1812966 (PMC13248018; doi:10.3389/frph.2026.1812966)
Supplement: Supplementary file 1 [file Supplementaryfile1.pdf]

Reproductive Health, STIs/RTIs and vaccine Acceptability Questionnaire in India

This questionnaire contains statements concerning General health including Reproductive health, RTI symptoms , lifestyle and health literacy.

Reproductive tract infections (RTIs) are a substantial economic burden and public health concern in developing South Asian nations, leading to reproductive morbidity and unfavoruable reproductive health problems in both men and women.

We are launching a survey on RTI symptoms burden, knowledge, attitude and perception towards RTIs/STIs , and STIs vaccines acceptability . We wish to ascertain the status of your general health and if you had/have any medical complaints that you wish to divulge.

We invite participants (above 18 years old) to participate in this study. It has no unexpressed agenda of violating anybody's private space and is completely academic and research   oriented. Participation in the study is completely voluntary.

There are no foreseeable risks associated with this study. If you decide to participate in this study, your identity and the information shared by you will be kept completely confidential to the full extent of law. All the investigators in this project would be legally bound to keep your details and information strictly confidential. The list connecting your name to this number will be kept in a locked file and only the principal investigator and involved researcher will have access to the survey you participated in. The survey would take 8-10 minutes of your precious time. Please select the appropriate response for all questions. There are no right or wrong answers. All responses will be completely anonymous. It is important that you try to answer ALL the questions for statistical convenience. However, if you feel uncomfortable answering any questions, you can skip that question or withdraw from the survey at any point. We respect your privacy and assure you that some sensitive questions are only being asked because they are relevant to this study.   If you have any questions about this study, please contact the concerned investigators.

Respondents would not be given any remuneration or incentives for participation and may reply only once to the survey questionnaire.

Thank you for your participation

* Indicates required question

1. Email *

____

2. Course name and Year *

____

3. I have been explained the details of the research study and give my consent to participate in this study. *

Mark only one oval.

- ( ) Yes
- ( ) No

4. Life stage *

Check all that apply.

- [ ] Unmarried
- [ ] Married

5. Age

____

6. Weight in kg and Height in cm/feet *

____

7. What is the highest level of education you have completed? *

Mark only one oval.

- ( ) 8th grade or less
- ( ) 10th grade
- ( ) 12th grade
- ( ) Graduate
- ( ) Post Graduate and above

8. Occupation *

Mark only one oval.

- Academician/Government/Private Sector/Healthcare Workers
- Non teaching staff
- College Student
- Other: ____________________

9. Have you taken any medicine prescribed by a doctor in the last 3 months (for RTI/UTI symptoms) *

Mark only one oval.

- Yes
- No
- Not applicable
- self medication

Reproductive Health

Reproductive health plays a crucial role in overall well-being and specifically
in the capability to reproduce and deliver healthy babies in young women. Ensuring access to
adequate information, services, and resources related to reproductive health are essential for
improving health, sexual and social relationships, psychological well-being, and preventing reproductive tract infections (RTIs).

10. When did you attain menarche? (First period)? *

Mark only one oval.

- Before 10 year of age
- 10-12 year of age
- 12-16 year of age
- After 16 years of age
- Not applicable

11. How would you describe your Menstrual Cycle? *

Mark only one oval.

- Irregular (less than 8 total cycles per year)
- Regular (greater than 8 cycles per year)
- Not applicable

12. Please mark the length of menstrual cycle *

Mark only one oval.

- More than 21 days but less than 35 days
- More than 35 days but less than 45 days
- More than 45 days
- Not applicable
- Other: ____________________

13. Does any member of your family suffer from/ever suffered from any of the following conditions? (more than one option may be marked). *

Check all that apply.

- [ ] Diabetes
- [ ] Hypertension
- [ ] Miscarriage
- [ ] Cancer
- [ ] Cardiovascular disease
- [ ] Polycystic Ovarian disease
- [ ] Endometriosis
- [ ] Don't know
- [ ] Other: ____________________

14. Are you suffering from any of the following chronic conditions? (more than one option may be marked). *

Check all that apply.

- [ ] COVID-19
- [ ] Tuberculosis
- [ ] HIV
- [ ] Infertility
- [ ] Thyroid problem
- [ ] Hormonal Imbalance
- [ ] Polycystic Ovarian Disease
- [ ] Not applicable
- [ ] Other: ____________________

15. Have you ever suffered from any of these symptoms? (please select all that apply) *

Check all that apply.

- [ ] Lower abdominal pain
- [ ] Perianal pain (pain in and around anal/rectal region)
- [ ] Urinary tract infections
- [ ] Dysuria(painful/burning)urination
- [ ] Polyuria (frequent urination)
- [ ] Vaginal discharge and/or Itching
- [ ] Abnormal growth or mass in genital area
- [ ] Dyspareunia (painful intercourse)
- [ ] Vaginal discharge with odour
- [ ] Urinary problem after sexual contact
- [ ] Post Coital (intercourse) bleeding
- [ ] Backache
- [ ] Genital Ulceration
- [ ] Other: ______________________

16. Have you ever been diagnosed with any disease of reproductive tract (in the past 3 months)?
(please select all that apply) *

Check all that apply.

- [ ] Vaginitis (Vagina)
- [ ] Cervictis (Cervix)
- [ ] Pelvic inflammatory disease (endometrium, fallopian tube, ovaries)
- [ ] Others (Vulval infection, Bartholin gland infection)
- [ ] Not applicable

17. Personal hygiene behaviour (please select all that apply) *

Check all that apply.

- [ ] Private part cleaning
- [ ] Use of sanitary pads
- [ ] Use of cotton underwear
- [ ] None of these

18. Are you aware of methods of contraception? If Yes, which one (contraceptive) do you prefer?

Mark only one oval.

- Not aware
- Barrier method (Condoms)
- Contraceptive devices (Copper T, Intra uterine devices-IUD)
- Hormonal (MalA-D. Saheli etc)
- Emergency contraceptive
- Other: ____________________

19. Have you ever been diagnosed with STIs/RTIs? If yes, were you told by your doctor/nurse that your STIs/RTIs was: *

Mark only one oval.

- Not ever diagnosed with STI/RTI
- Gonorrhea
- Chlamydia
- Syphilis
- HIV or AIDS
- Hepatitis B
- Candidiasis
- Bacterial vaginosis
- Trichomoniasis
- Genital herpes
- Other: ____________________

Knowledge, Hesitancy and Acceptance of vaccines

Vaccines are used to protect you against infections before you are exposed to them. Many vaccines are given to infants or young children, while others are given at older ages including adolescents and adults. There are two STI vaccines that are currently available: the human papillomavirus (HPV) vaccine (in BC, administered in schools to adolescents) and the Hepatitis B vaccine (typically administered to infants or children). Some STIs such as syphilis, chlamydia, gonorrhoea and trichomoniasis can be treated and cured with antibiotics, but if you have unprotected sex, you can get it again. In other words, being diagnosed and treated once for these STIs does not mean you are protected against subsequent infection with the same microorganism. Other STIs such as the herpes virus and HIV are chronic conditions that cannot be cured but can be managed upon diagnosis.

20. Have you heard of HPV vaccines? *

Mark only one oval.

- Yes
- No

21. Have you ever received HPV vaccines? *

Mark only one oval.

- Yes
- No

22. If yes, at what age did you receive your first vaccination for HPV? *

____

23. Have you heard of Hepatitis B vaccine?

Mark only one oval.

- Yes
- No

24. Have you ever received the Hepatitis B vaccine? *

Mark only one oval.

- Yes
- No

25. If yes, at what age did you receive your first vaccination for Hepatitis B vaccine? *

____

26. Rate your understanding about STI ? *

Mark only one oval.

- Good understanding
- Poor understanding
- Moderate understanding

27. If available today, would you be interested in receiving a vaccine to prevent STIs/RTIs ? *

Mark only one oval.

- Not Interested
- Neutral
- Interested

28. If and when available, which of the following STI vaccines would you be interested in receiving? (You can check more than one answer) *

Check all that apply.

- [ ] Syphilis
- [ ] Chlamydia
- [ ] Gonorrhea
- [ ] Herpes
- [ ] HIV
- [ ] Trichomoniasis
- [ ] Not Interested

29. What would motivate you to receive an STI vaccine? (please select all that apply) *

Check all that apply.

- [ ] To protect myself against STIs
- [ ] To protect my partner(s) from an undiagnosed STI/RTI
- [ ] Lower the need to treat recurring infections using medications
- [ ] Fewer visits to the doctor or clinic
- [ ] If pregnant, to protect my unborn child from infection
- [ ] I would use condoms less if I received an STI/RTI vaccine
- [ ] Because I belong to high risk category
- [ ] Other: ______________________

30. Please reflect on all your reason(s) for not being interested in receiving an STI vaccination. *

Check all that apply.

- [ ] Cost of the vaccine if it was not publicly funded
- [ ] Distance or convenience of locating a clinic to get vaccine
- [ ] Privacy (having the vaccination on my health record)
- [ ] I am unsure of the protection or benefits of the vaccine
- [ ] I worry about new vaccines being unsafe
- [ ] I would be embarassed about getting a vaccine for an STI
- [ ] I might be judged for getting a vaccine for an STI
- [ ] I have low immunity, older age or comorbidity
- [ ] I do not believe it will be a solution for STI
- [ ] Unavailability of STI vaccine(s)

31. Please reflect on where you would like to receive information about STI vaccines. *

Check all that apply.

- [ ] In person conversations with physician
- [ ] In person conversations with a public health nurse
- [ ] Trusted Organization (ICMR, WHO, CDC and UBC)
- [ ] Brochure in clinics
- [ ] Online resources and social media (X, Instagram, reddit , facebook etc)
- [ ] Government advertisements Other than these
- [ ] Trusted family members or friends
- [ ] Public advertisements (Eg. public transit posters)
- [ ] Other: ____________________

32. If STI vaccines were available, where would you prefer to get vaccinated? *

Mark only one oval.

- STI clinics
- Doctor's clinics/practices
- Walk in clinics
- Pharmacies
- School vaccination programs
- Hospitals
- Community camps
- Government camps

33. Would you be willing to pay to receive an STI vaccination? *

Mark only one oval.

- Yes
- No
- Maybe

34. IF STI vaccines were available, when do you think it would be best to first offer STI vaccines? *

Mark only one oval.

- Infancy
- Childhood
- Adolescence
- Early adulthood
- Adulthood
- Late adulthood

35. Do you think STIs vaccination could be an effective way to prevent STIs *

Mark only one oval.

- Strongly disagree
- Disagree
- Neutral
- Agree
- Strongly agree

36. If an STI vaccine were available, would you encourage your partner(s) to get immunized ? *

Mark only one oval.

- Strongly disagree
- Disagree
- Neutral
- Agree
- Strongly agree

37. If an STI vaccine were available, would you get immunized to prevent STI transmission to your child during birth or pregnancy? *

Mark only one oval.

- Strongly disagree
- Disagree
- Neutral
- Agree
- Strongly agree

38. Which of the following would be helpful to accept STI vaccine(s)? *

Mark only one oval.

- ( ) Will wait to observe other people’s reaction to vaccine
- ( ) Detailed review about vaccine’s adverse events and constituents
- ( ) if doctor or healthcare would recommend
- ( ) Other: ______________________

This content is neither created nor endorsed by Google.

Google Forms
