# Supplementary material for: Prevalence, risk factors, and perceptions of vaccination against reproductive tract infections among urban females in Delhi: a cross-sectional study
Source: Front Reprod Health. 2026 May 26;8:1812966. doi: 10.3389/frph.2026.1812966 (PMC13248018; doi:10.3389/frph.2026.1812966)
Supplement: Supplementary file 2 [file Table1.docx]

**Supplementary Table 1: History of Comorbidities Associated with Symptoms Suggestive of Reproductive Tract Infections Among Urban Females**

| **Variables** | **RTI absent**  **N (%)**  **679 (35.4)** | **Low risk RTI**  **N (%)**  **446 (23.2)** | **High risk RTI**  **N (%)**  **795 (41.4)** | **Low risk RTI**  **Vs RTI absent**  **χ2**  ***P-value*** | **High risk RTI**  **Vs RTI absent**  **χ2**  ***P-value*** | **Low risk RTI**  **Vs High risk RTI**  **χ2**  ***P-value*** |
| --- | --- | --- | --- | --- | --- | --- |
| **Family history of comorbidities** | | |  |  |  |  |
| Yes  No | 352 (51.8)  327 (48.2) | 261 (58.5)  185 (41.5) | 541 (68.1)  254 (31.9) | 4.577  0.032 | 39.618  0.000** | 10.938  0.001** |
|  |  |  |  |  |  |  |
| **Existing medical conditions** | | |  |  |  |  |
| Yes  No | 164 (24.8)  497 (75.2) | 96 (22.4)  333 (77.6) | 197 (25.7)  570 (74.3) | 0.719  0.396 | 0.101  0.751 | 1.453  0.228 |

*98.33% confidence intervals were calculated after correction to significance level using Bonferroni method. RTI, reproductive tract infection.
