# Supplementary material for: Prevalence, risk factors, and perceptions of vaccination against reproductive tract infections among urban females in Delhi: a cross-sectional study
Source: Front Reprod Health. 2026 May 26;8:1812966. doi: 10.3389/frph.2026.1812966 (PMC13248018; doi:10.3389/frph.2026.1812966)
Supplement: Supplementary file 3 [file Table2.docx]

**Supplementary Table 2: Association of HPV and Hepatitis B Vaccination Status with Symptoms Suggestive of Reproductive Tract Infections Among Females**

| **Variables** | **RTI absent**  **N (%)**  **679 (35.4)** | **Low risk RTI**  **N (%)**  **446 (23.2)** | **High risk RTI**  **N (%)**  **795 (41.4)** | **Low risk RTI**  **Vs RTI absent**  **χ2**  ***P-value*** | **High risk RTI**  **Vs RTI absent**  **χ2**  ***P-value*** | **Low risk RTI**  **Vs High risk RTI**  **χ2**  ***P-value*** |
| --- | --- | --- | --- | --- | --- | --- |
| **HPV Vaccination Status** | | |  |  |  |  |
| Yes  No | 49 (7.2)  629 (92.8) | 46 (10.3)  400 (89.7) | 63 (7.9)  732 (92.1) | 2.952  0.086 | 0.170  0.680 | 1.749  0.186 |
|  |  |  |  |  |  |  |
| **Hepatitis B Vaccination Status** | | |  |  |  |  |
| Yes  No | 161 (23.7)  517 (76.3) | 130 (29.1)  316 (70.9) | 206 (25.9)  589 (74.1) | 3.815  0.051 | 0.805  0.370 | 1.356  0.244 |

*98.33% confidence intervals were calculated after correction to significance level using Bonferroni method. RTI, reproductive tract infection.
