# Supplementary material for: Prevalence, risk factors, and perceptions of vaccination against reproductive tract infections among urban females in Delhi: a cross-sectional study
Source: Front Reprod Health. 2026 May 26;8:1812966. doi: 10.3389/frph.2026.1812966 (PMC13248018; doi:10.3389/frph.2026.1812966)
Supplement: Supplementary file 4 [file Table3.docx]

**Supplementary Table 3: Variance Inflation Factors (VIF) for Predictors in the Multinomial Logistic Regression Model**

| **Variables** | **VIF** | **Tolerance (1/VIF)** |
| --- | --- | --- |
| **Age (Years)** | 2.221 | 0.450 |
| **Marital Status** | 2.216 | 0.451 |
| **Prescription Medication Use (last 3 months)** | 1.032 | 0.969 |
| **Age at Menarche (First Period)** | 1.017 | 0.983 |
| **Family History of Medical Conditions** | 1.029 | 0.972 |
| **Menstrual Hygiene Practices** | 1.022 | 0.979 |
| **Awareness of Contraceptive Methods** | 1.025 | 0.976 |
| **Prior Diagnosis of STIs/RTIs** | 1.024 | 0.976 |

*Note: VIF < 5 is considered acceptable (no multicollinearity concern). All values here are well below this threshold, confirming the independence of predictors in the final model.*
