# Supplementary material for: Prevalence, risk factors, and perceptions of vaccination against reproductive tract infections among urban females in Delhi: a cross-sectional study
Source: Front Reprod Health. 2026 May 26;8:1812966. doi: 10.3389/frph.2026.1812966 (PMC13248018; doi:10.3389/frph.2026.1812966)
Supplement: Supplementary file 5 [file Table4.docx]

**Supplementary Table 4: Sensitivity Analyses- Menstrual Hygiene and Contraceptive Awareness ORs Across Restricted Samples (High-Risk RTI Model)**

| **Analysis** | **Hygiene**  **OR** | **95% CI** | ***P-value*** | **Contraceptive Awareness**  **OR** | **95% CI** | ***P-value*** |
| --- | --- | --- | --- | --- | --- | --- |
| **Full sample (N=1,920)** | 3.183 | [1.668–6.072] | < 0.001** | 1.919 | [1.380–2.669] | < 0.001** |
| **Excluding recent medication users (N=1,523)** | 2.210 | [0.959–5.090] | 0.063 | 1.780 | [1.217–2.603] | 0.003** |
| **Additionally excluding prior RTI diagnosis (N=1,486)** | 1.870 | [0.710–4.940] | 0.203 | 1.610 | [1.091–2.376] | 0.016* |

*OR = odds ratio. 'Hygiene' = Menstrual Hygiene Practices; 'Contra.' = Contraceptive Method Awareness. Restriction 1: excludes participants who used RTI/UTI medication in the past 3 months (N=397 excluded). Restriction 2: additionally excludes participants with a prior RTI diagnosis (further N=37 excluded). Attenuation of the hygiene OR across restrictions supports reverse causation as a major explanation for the main analysis finding.*
