# Supplementary figures and images for: Prevalence, risk factors, and perceptions of vaccination against reproductive tract infections among urban females in Delhi: a cross-sectional study
Source: Front Reprod Health. 2026 May 26;8:1812966. doi: 10.3389/frph.2026.1812966 (PMC13248018; doi:10.3389/frph.2026.1812966)

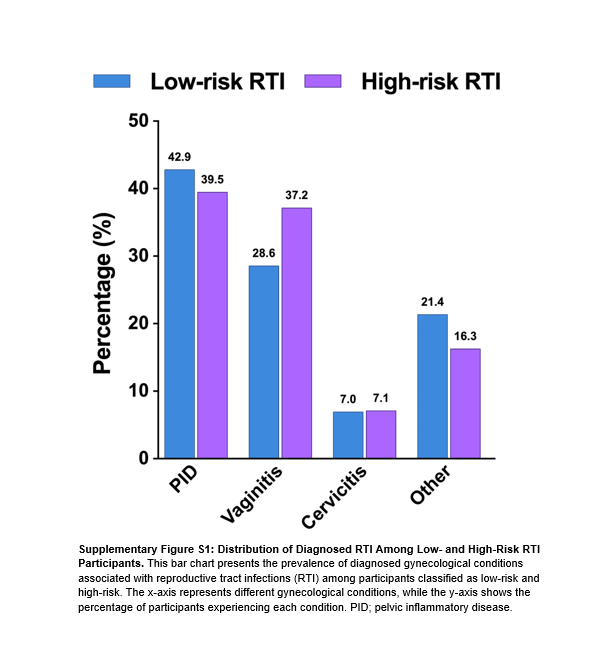

Supplement: Supplementary file 6 [file Image1.png]

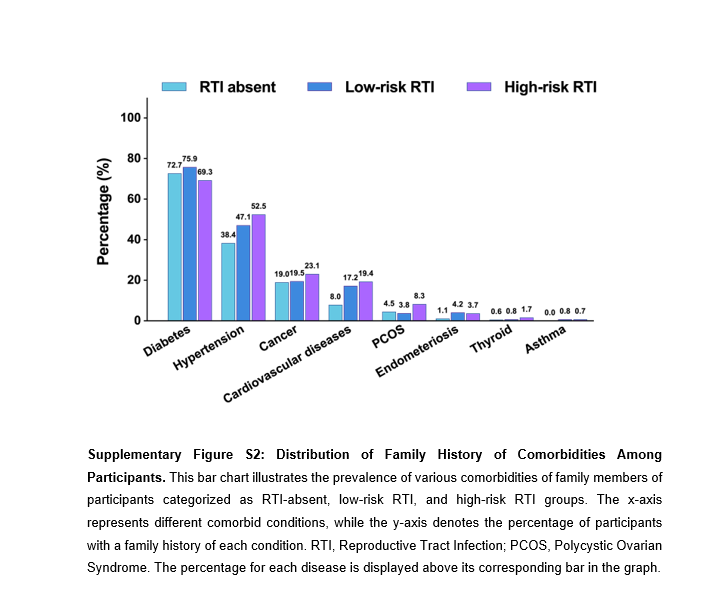

Supplement: Supplementary file 7 [file Image2.png]
